# Supplementary material for: Genomic adaptation to small population size and saltwater consumption in the critically endangered Cat Ba langur
Source: Nat Commun. 2024 Oct 2;15:8531. doi: 10.1038/s41467-024-52811-7 (PMC11447269; doi:10.1038/s41467-024-52811-7)
Supplement: Supplementary file 1 — Supplementary Information [file 41467_2024_52811_MOESM1_ESM.pdf]

Supplementary information for:

Genomic adaptation to small population size and saltwater consumption in the critically endangered Cat Ba langur

Liye Zhang[1,2,3,*], Neahga Leonard[4], Rick Passaro[4], Mai Sy Luan[4], Pham Van Tuyen[4], Le Thi Ngoc Han[4], Nguyen Huy Cam[4], Larry Vogelnest[5], Michael Lynch[6], Amanda E. Fine[7], Nguyen Thi Thanh Nga[8], Nguyen Van Long[8], Benjamin M. Rawson[9], Alison Behie[10], Nguyen Van Truong[1,11,12], Minh D. Le[12,13], Tilo Nadler[14], Lutz Walter[1], Tomas Marques-Bonet[15,16,17,18], Michael Hofreiter[11,*], Ming Li[3,*], Zhijin Liu[19,*], and Christian Roos[1,20,*]

[1]Primate Genetics Laboratory, German Primate Center, Leibniz Institute for Primate Research, Göttingen, Germany

[2]International Max Planck Research School for Genome Science (IMPRS-GS), University of Göttingen, Göttingen, Germany

[3]CAS Key Laboratory of Animal Ecology and Conservation Biology, Institute of Zoology, Chinese Academy of Sciences, Beijing, China

[4]Cat Ba Langur Conservation Project (CBLCP), Cat Ba National Park, Cat Ba Island, Cat Hai District, Hai Phong Province, Vietnam

[5]Taronga Conservation Society Australia, Mosman, NSW, Australia

[6]Melbourne Zoo, Zoos Victoria, Parkville, VIC, Australia

[7]Wildlife Conservation Society (WCS), Health Program, New York, NY, USA

[8]Wildlife Conservation Society (WCS), Vietnam Country Program, Hanoi, Vietnam

[9]World Wildlife Fund for Nature (WWF) International, Gland, Switzerland

[10]School of Archaeology and Anthropology, The Australian National University, Canberra, ACT, Australia

[11]Evolutionary Adaptive Genomics, Institute of Biochemistry and Biology, Department of Science, University of Potsdam, Potsdam, Germany

[12]Central Institute for Natural Resources and Environmental Studies, Vietnam National University, Hanoi, Vietnam

[13]Faculty of Environmental Sciences, University of Science, Vietnam National University, Hanoi, Vietnam

[14]Three Monkeys Wildlife Conservancy, Nho Quan District, Ninh Binh Province, Vietnam

[15]Institute of Evolutionary Biology (UPF-CSIC), PRBB, Barcelona, Spain

[16]Catalan Institution of Research and Advanced Studies (ICREA), Barcelona, Spain

[17]CNAG-CRG, Centre for Genomic Regulation (CRG), Barcelona Institute of Science and

Technology (BIST), Barcelona, Spain
[18]Institut Català de Paleontologia Miquel Crusafont, Universitat Autònoma de Barcelona, Edifici ICTA-ICP, 08193 Cerdanyola del Vallès, Spain
[19]College of Life Sciences, Capital Normal University, Beijing, China
[20]Gene Bank of Primates, German Primate Center, Leibniz Institute for Primate Research, Göttingen, Germany

[*] Authors to whom any correspondence should be addressed.
Liye Zhang (lzhang@dpz.eu), Michael Hofreiter (michael.hofreiter@uni-potsdam.de), Ming Li (lim@ioz.ac.cn), Zhijin Liu (6888@cnu.edu.cn), Christian Roos (croos@dpz.eu)

This PDF file includes:

Supplementary Figures 1 to 17

Supplementary Tables 1 to 11

Other supplementary information for this manuscript includes the following:

Supplementary Movie 1 (.mp4)

Supplementary Data 1 to 10 (.xlsx)

Source Data (.xlsx)

Supplementary Table 1. Mapping statistics for the four *T. poliocephalus* individuals (Tfra_2.0 reference genome).

Samples	Clean reads	Clean kilobases	Mapped reads	Mapped kilobases	Mapping rate	Read depth
Tpol1	827,229,192	124,084,379	822,996,026	122,895,861	99.49%	36.52
Tpol2	632,341,386	94,851,208	627,331,764	93,552,390	99.21%	28.78
Tpol3	766,848,856	115,027,328	762,030,874	113,612,134	99.37%	33.23
Tpol4	719,690,908	107,953,636	715,839,944	106,893,419	99.46%	32.26

Supplementary Table 2. Kinship coefficient for the four *T. poliocephalus* individuals (ID1: Individual ID for the first individual of the pair, ID2: Individual ID for the second individual of the pair, N_SNP: The number of SNPs that do not have missing genotypes in either of the individual, HetHet: Proportion of SNPs with double heterozygotes (e.g., AG and AG), IBS0: Proportion of SNPs with zero IBS (identical-by-state) (e.g., AA and GG), Kinship: Estimated kinship coefficient from the SNP data), A negative kinship coefficient indicates an unrelated relationship.

ID1	ID2	N_SNP	HetHet	IBS0	Kinship
Tpol1	Tpol2	84479537	0.0029	0.0072	-1.0776
Tpol1	Tpol3	84477693	0.0029	0.0078	-1.2430
Tpol1	Tpol4	84479881	0.0028	0.0072	-1.0872
Tpol2	Tpol3	84477650	0.0031	0.0078	-1.1388
Tpol2	Tpol4	84479604	0.0028	0.0074	-1.0924
Tpol3	Tpol4	84479285	0.0031	0.0071	-1.0668

Supplementary Table 3. Overview about methods and mapping data used for specific analyses.

Analysis	Reference genome	Method or software
Phylogeny	Mmul_10	NJ and ML
PCA	Mmul_10	EIGENSOFT and *frappe*
Population structure	Mmul_10	Admixture
Gene flow	Mmul_10	*D*-statistics, qpDstat, Dtrios and Dinvestigate
Genetic diversity	Mmul_10	Vcftools, with 50kb non-overlapping windows
Runs of homozygosity	Tfra_2.0	PLINK, bcftools, with max. 1 heterozygous and 50 missing calls per window
Deleterious mutation	Mmul_10	snpEFF
Genetic load	Mmul_10	GERP (≥4.0)
Positive selection	Tfra_2.0	XP-EHH, θπ, Ka/Ks
Non-synonymous variants	Tfra_2.0	snpEFF

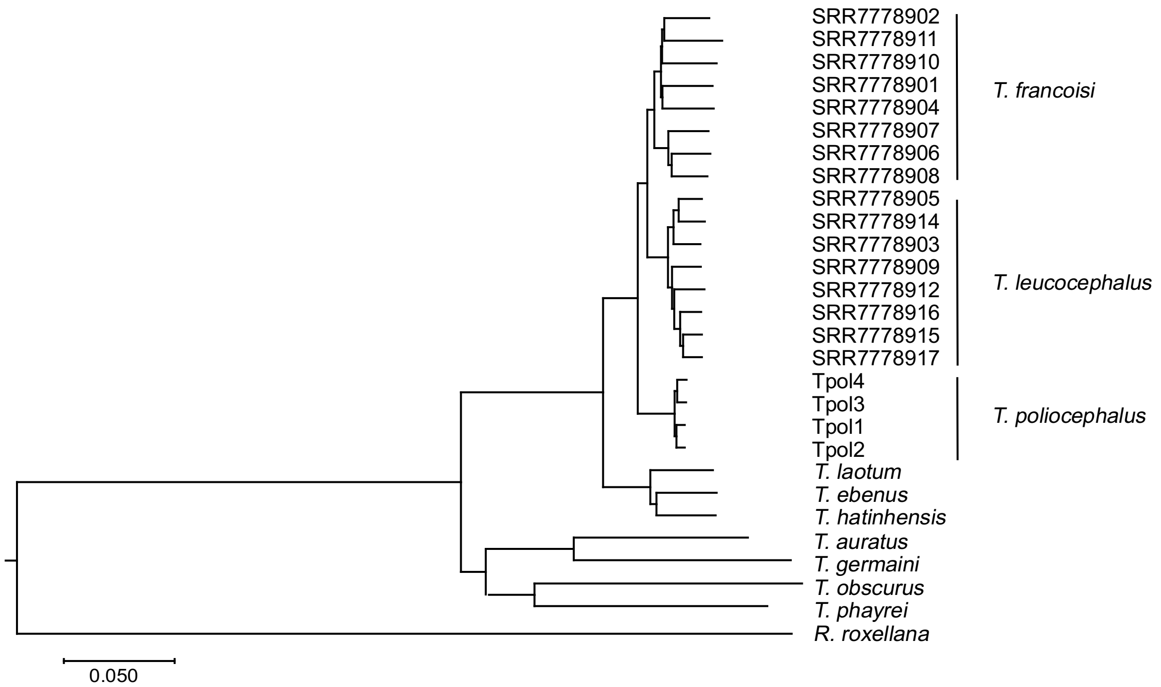

Supplementary Figure 1. Neighbor-joining tree with all 27 *Trachypithecus* individuals based on autosomal SNP data (Mmul_10 reference genome). The tree is rooted with *R. roxellana*. All nodes are supported by bootstrap values of 100%. The bar indicates substitutions per site.

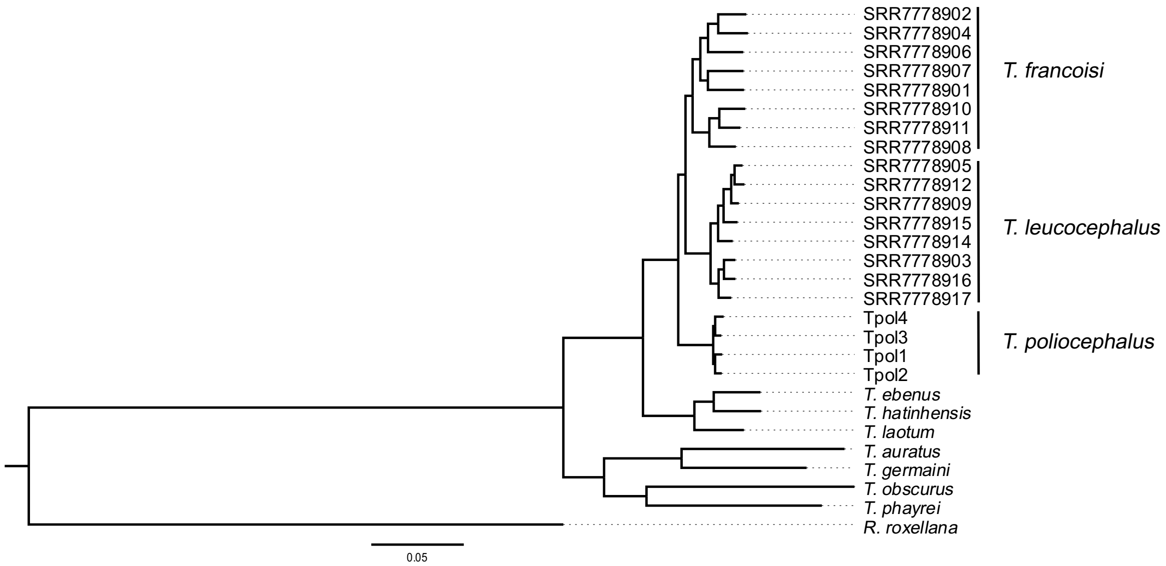

Supplementary Figure 2. Maximum-likelihood tree with all 27 *Trachypithecus* individuals based on autosomal SNP data (Mmul_10 reference genome). The tree is rooted with *R. roxellana*. All nodes are supported by bootstrap values of >95%. The bar indicates substitutions per site.

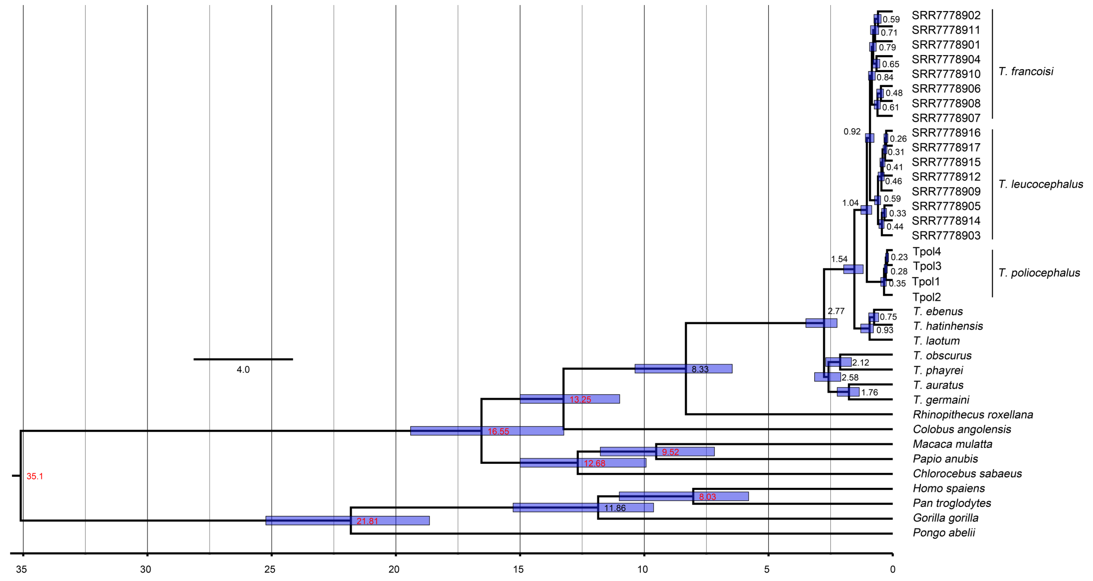

Supplementary Figure 3. Ultrametric tree showing phylogenetic relationships and divergence times among investigated langurs and various outgroup species (Mmul_10 reference genome). Numbers at nodes refer to million years ago and the blue bars indicate 95% confidence intervals.

Supplementary Table 4. Estimated divergence times and 95% confidence intervals (CI), both in million years ago (nodes used for calibration are marked in red).

Node	Divergence time	95% CI
Cercopithecoidea – Hominoidea	35.10	-
Homininae – Ponginae	21.81	18.65-25.23
Cercopithecinae – Colobinae	16.55	13.24-19.41
Colobini – Presbytini	13.25	10.99-15.00
Cercopithecini – Papionini	12.68	9.93-15.00
Homo & *Pan* – *Gorilla*	11.86	9.63-15.28
Macaca – *Papio*	9.52	7.18-11.77
Rhinopithecus – *Trachypithecus*	8.33	6.46-10.37
Homo – *Pan*	8.03	5.80-11.01
T. francoisi group – *T. obscurus* & *T. cristatus* groups	2.77	2.24-3.50
T. obscurus group – *T. cristatus* group	2.58	2.09-3.14
T. obscurus – *T. phayrei*	2.12	1.66-2.70
T. auratus – *T. germaini*	1.76	1.35-2.24
southern – northern limestone langurs	1.54	1.19-1.97
T. francoisi & *T. leucocephalus* – *T. poliocephalus*	1.04	0.84-1.28
T. ebenus & *T. hatinhensis* – *T. laotum*	0.93	0.78-1.29
T. francoisi – *T. leucocephalus*	0.92	0.75-1.10
T. ebenus – *T. hatinhensis*	0.75	0.57-0.97

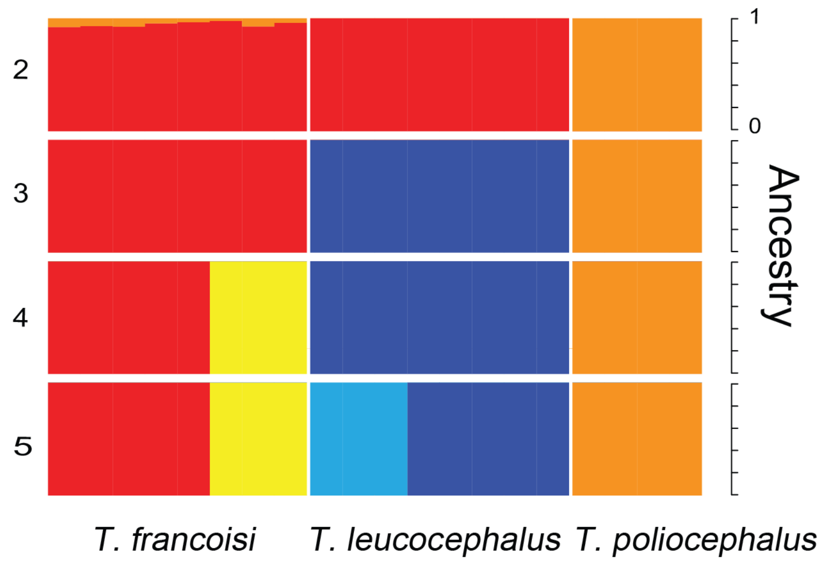

Supplementary Figure 4. Admixture plots for eight *T. francoisi*, eight *T. leucocephalus* and four *T. poliocephalus* individuals. Plots are shown for $K = 2–5$ with $K = 3$ as the most suitable population structure (cross-validation error: $K = 2$: 0.70556, $K = 3$: 0.69680, $K = 4$: 0.84417, $K = 5$: 0.93614) (Mmul_10 reference genome).

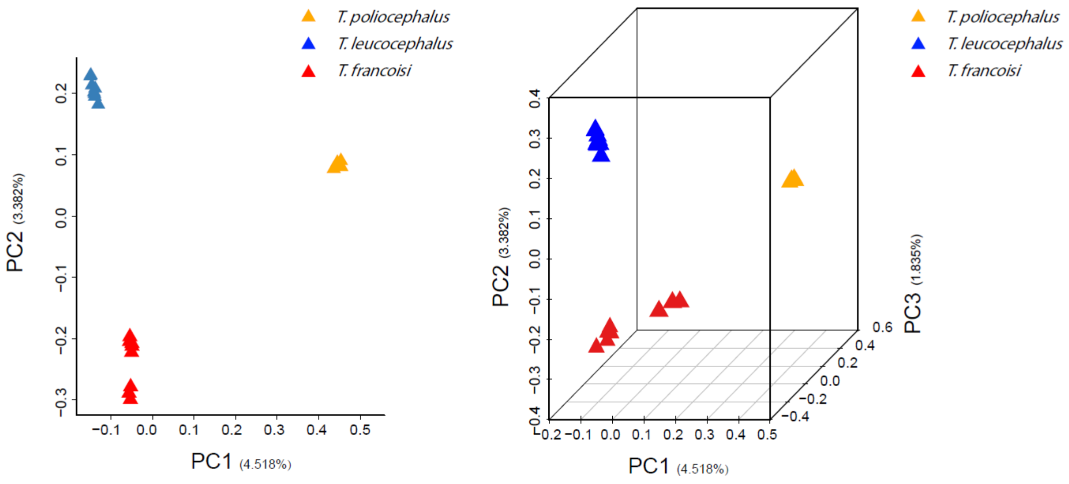

Supplementary Figure 5. Plots of the first two (left) and three (right) components among eight *T. francoisi*, eight *T. leucocephalus* and four *T. poliocephalus* individuals. The fraction of the variance explained is 4.518% for PC1, 3.382% for PC2 and 1.835% for PC3 (for Tracy Widom statistics and *p*-values see Supplementary Table 5) (Mmul_10 reference genome).

Supplementary Table 5. Tracy-Widom (TW) statistics and *p*-values for the first ten eigenvalues in the PCA (see Supplementary Figure 5).

#N	Eigenvalue	Difference	TW	*p*-value	Effect. n
1	4.517856	NA	1.048	0.045395	17.017
2	3.382109	-1.13575	2.291	0.006305	22.44
3	1.835445	-1.54666	0.807	0.063296	41.101
4	1.224004	-0.61144	-1.762	0.655818	51.783
5	0.989743	-0.23426	-3.146	0.946648	51.563
6	0.951196	-0.03855	-2.782	0.899959	47.329
7	0.936657	-0.01454	-2.043	0.737665	43.595
8	0.922132	-0.01453	-1.012	0.419816	41.576
9	0.847420	-0.07471	-0.112	0.189203	43.909
10	0.733084	-0.11434	1.068	0.044127	55.408

Supplementary Table 6. Results of model testing with qpDstat (Mmul_10 reference genome).

O	P_1	P_2	P_3	D-score	Z-score	BABA	ABBA	Total
Rrox	*Tleu*	*Tfra*	*Tpol*	-0.0112	-2.881	481,455	492,380	74,369,547

*BABA model shows that O and P_2 share the same allele polymorphisms or P_1 and P_3 share the same allele polymorphisms. ABBA model means O and P_3 share the same allele polymorphisms. X^2 test of BABA and ABBA model, $p = 0.740$. $D = (O - P_1)(P_2 - P_3)/(O + P_1 - 2O{*}P_1)(P_2 + P_3 - 2P_2{*}P_3)$. If the Z-score is +ve, gene flow occurred either between O and P_2 or between P_1 and P_3; if the Z-score is -ve, gene flow occurred either between O and P_3 or between P_1 and P_2.

Supplementary Table 7. Results of model testing with Dtrios (Mmul_10 reference genome).

P_1	P_2	P_3	O	D-statistic	*p*-value	f4-ratios
Tfra	*Tleu*	*Tpol*	*Rrox*	0.0380728	0	0.02312

*D = (nABBA - nBABA)/(nABBA + nBABA), the software orders each trio assuming that the correct tree is the one where the BBAA pattern is more common than the discordant ABBA and BABA patterns, which are assumed to result for example from introgression. In addition, P_1 and P_2 are ordered so that nABBA≥nBABA and, therefore D is never negative.

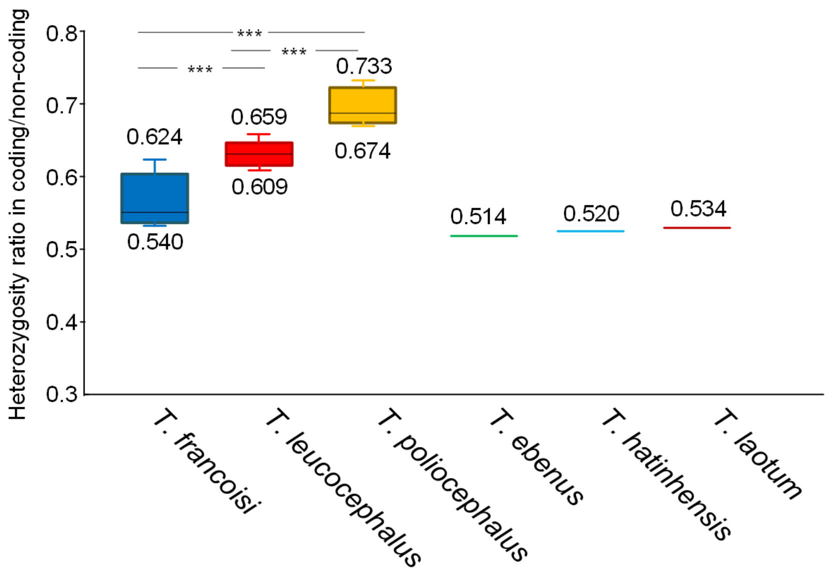

Supplementary Figure 6. Heterozygosity ratio in protein-coding versus non-protein-coding regions (One-way ANOVA test; n (*T. francoisi*) = 8, n (*T. leucocephalus*) = 8, n (*T. poliocephalus*) = 4; *** $p < 0.001$, $p_{(\mathrm{Tfra\text{-}Tleu})} = 0.00026$, $p_{(\mathrm{Tpol\text{-}Tfra})} = 0.000051$, $p_{(\mathrm{Tpol\text{-}Tleu})} = 0.00042$; all p values, minimum value, first quartile (Q1), median (Q2), third quartile (Q3), maximum value, interquartile range (IQR), lower whisker and upper whisker are provided in the Source Data file) (Mmul_10 reference genome).

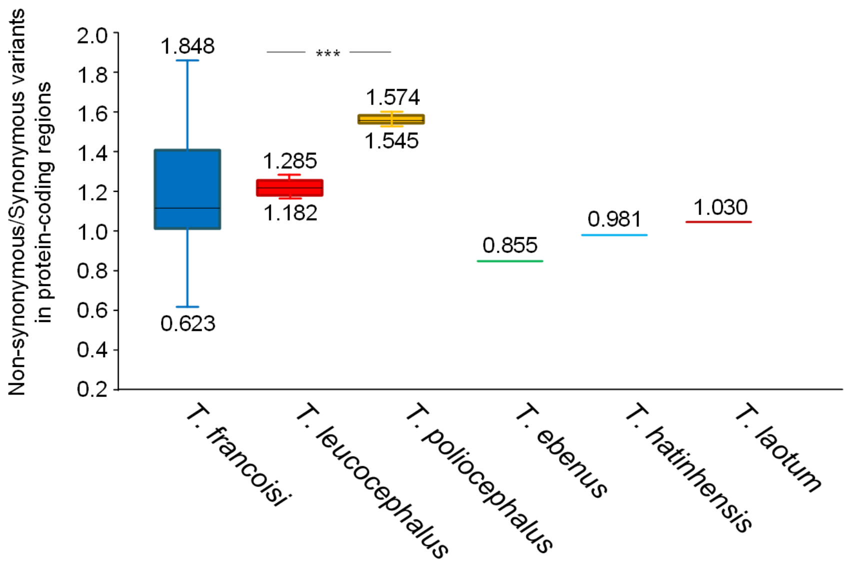

Supplementary Figure 7. Heterozygosity ratio of non-synonymous versus synonymous variants in protein-coding regions (One-way ANOVA test; *** $p < 0.001$, $p_{(\mathrm{Tpol\text{-}Tleu})} = 6.47\times10^{-9}$; all p values, minimum value, first quartile (Q1), median (Q2), third quartile (Q3), maximum value, interquartile range (IQR), lower whisker and upper whisker are provided in the Source Data file) (Mmul_10 reference genome).

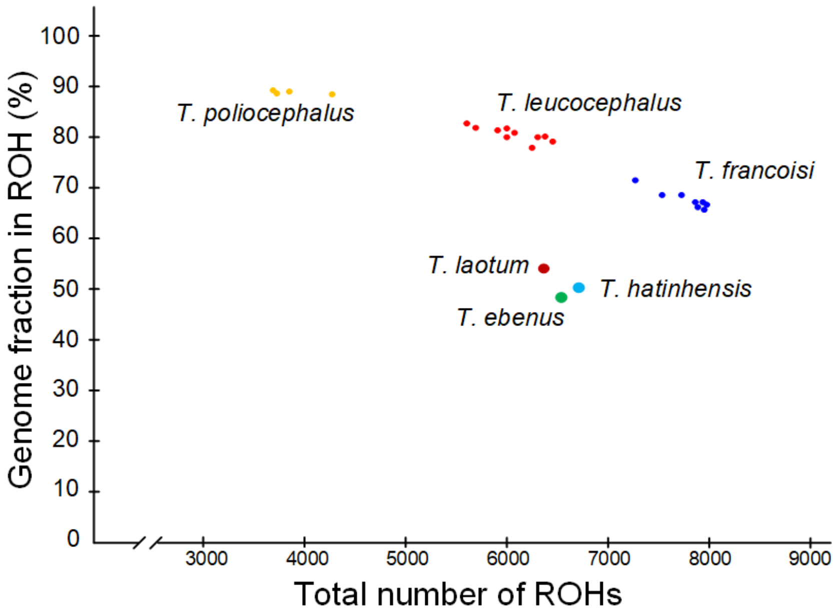

Supplementary Figure 8. Genome fraction in ROH vs the total number of ROHs (Tfra_2.0 reference genome).

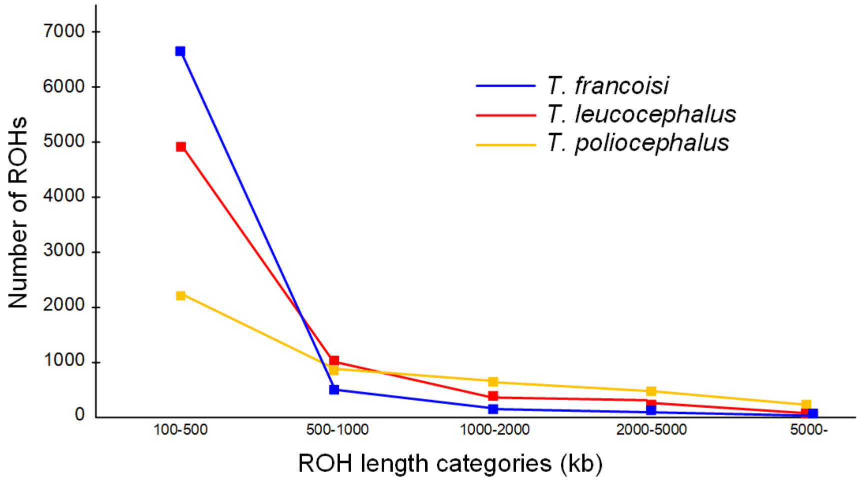

Supplementary Figure 9. Number of ROHs in different ROH length categories (Tfra_2.0 reference genome).

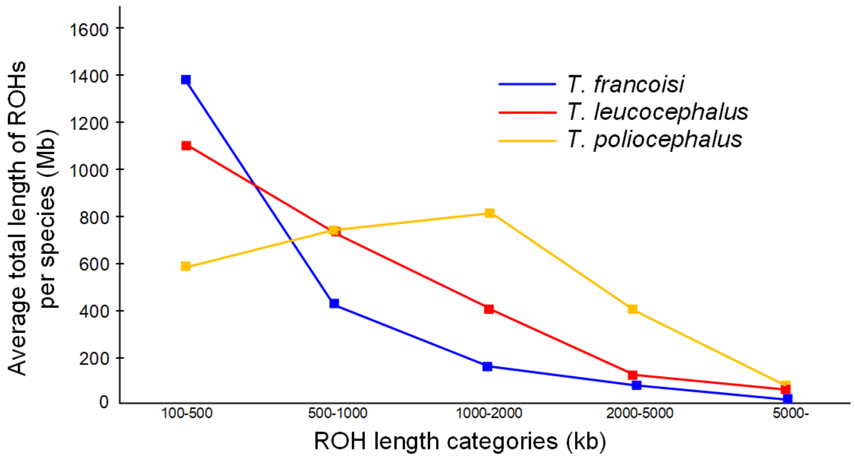

Supplementary Figure 10. Average total length of ROHs per species in different ROH length categories (Tfra_2.0 reference genome).

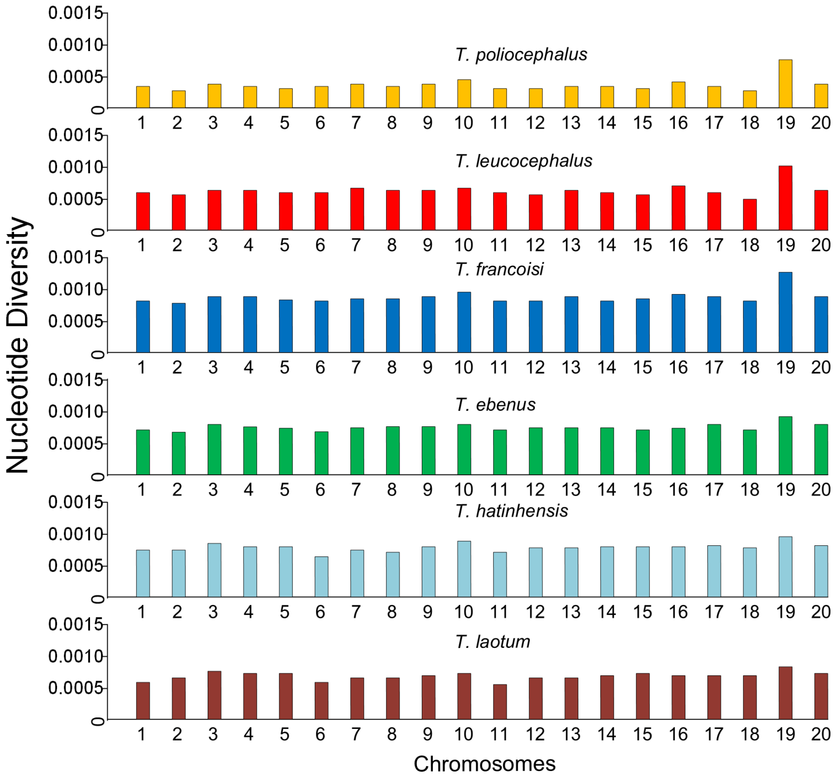

Supplementary Figure 11. Nucleotide diversity for individual autosomes in six limestone langur species. The difference between chr19 and other autosomes is significant in *T. poliocephalus* and *T. leucocephalus* (One-way ANOVA test; $p < 0.001$) (Mmul_10 reference genome).

Supplementary Table 8. Number and ratio of genes in ROHs for each chromosome in *T. poliocephalus*. The difference between chr19 and other autosomes is significant (One-way ANOVA test; $p < 0.001$) (Mmul_10 reference genome).

		ROH coverage threshold						
**CHR**	**Total_number**	**50%**	**60%**	**70%**	**80%**	**90%**	**100%**	**Ratio**
chr1	3227	2729	2710	2691	2660	2627	2530	0.7840
chr2	1793	1617	1602	1585	1565	1546	1474	0.8221
chr3	2004	1625	1612	1602	1591	1565	1503	0.7500
chr4	1870	1633	1621	1613	1598	1583	1526	0.8160
chr5	1385	1212	1204	1191	1177	1153	1096	0.7913
chr6	1573	1345	1334	1323	1307	1286	1230	0.7819
chr7	2324	1857	1844	1831	1807	1779	1717	0.7388
chr8	1238	1096	1089	1083	1071	1058	1012	0.8174
chr9	1299	1051	1041	1034	1023	1006	967	0.7444
chr10	1605	1277	1260	1249	1234	1222	1181	0.7358
chr11	1669	1455	1446	1438	1421	1406	1347	0.8071
chr12	1124	997	988	980	974	962	913	0.8123
chr13	1121	962	956	943	935	930	900	0.8028
chr14	1888	1567	1556	1543	1530	1503	1446	0.7659
chr15	1224	1009	1000	989	978	959	911	0.7443
chr16	1657	1329	1317	1310	1300	1288	1259	0.7598
chr17	693	608	607	604	597	591	561	0.8095
chr18	596	534	525	521	518	513	487	0.8171
chr19	1762	1198	1188	1178	1166	1152	1122	0.6368
chr20	1213	988	979	973	964	949	927	0.7642

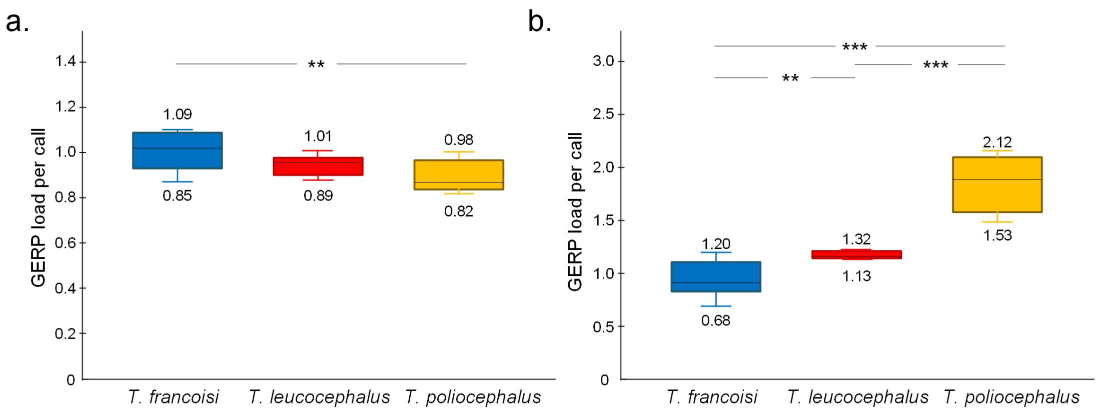

Supplementary Figure 12. Masked and realized load. Genetic load divided into the components (**a**) masked load and (**b**) realized load estimated as the sum of GERP scores over all deleterious derived alleles in heterozygous and homozygous genotypes, respectively, divided by the total number of calls in each individual (One-way ANOVA test; *** $p < 0.001$, ** $p < 0.05$; masked load: $p_{(Tpol\text{-}Tfra)} = 0.03771$, realized load: $p_{(Tpol\text{-}Tfra)} = 1.75\text{x}10^{-5}$, $p_{(Tpol\text{-}Tleu)} = 2.29\text{x}10^{-5}$, $p_{(Tfra\text{-}Tleu)} = 0.00101$; all p values, minimum value, first quartile (Q1), median (Q2), third quartile (Q3), maximum value, interquartile range (IQR), lower whisker and upper whisker are provided in the Source Data file) (Mmul_10 reference genome).

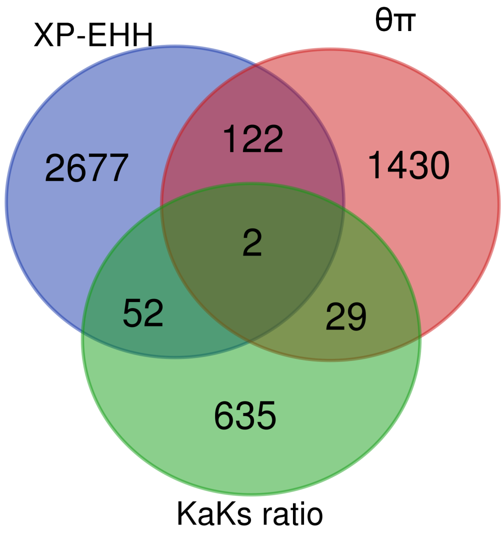

Supplementary Figure 13. Venn diagram showing results of the positive selection analysis using three methods.

Supplementary Table 9. Results of functional enrichment analyses related to calcium metabolism based on all genes with non-synonymous variants in *T. poliocephalus*.

#Term	Database	ID	Input	Background	*p*-value	Corrected *p*-value
calcium ion binding	GO term	GO:0005509	71	693	1.17E-13	4.06E-11
calcium-mediated signaling	GO term	GO:0019722	13	82	2.69E-05	0.001454285
calcium signaling pathway	KEGG pathway	hsa04020	16	193	0.002945935	0.045041411

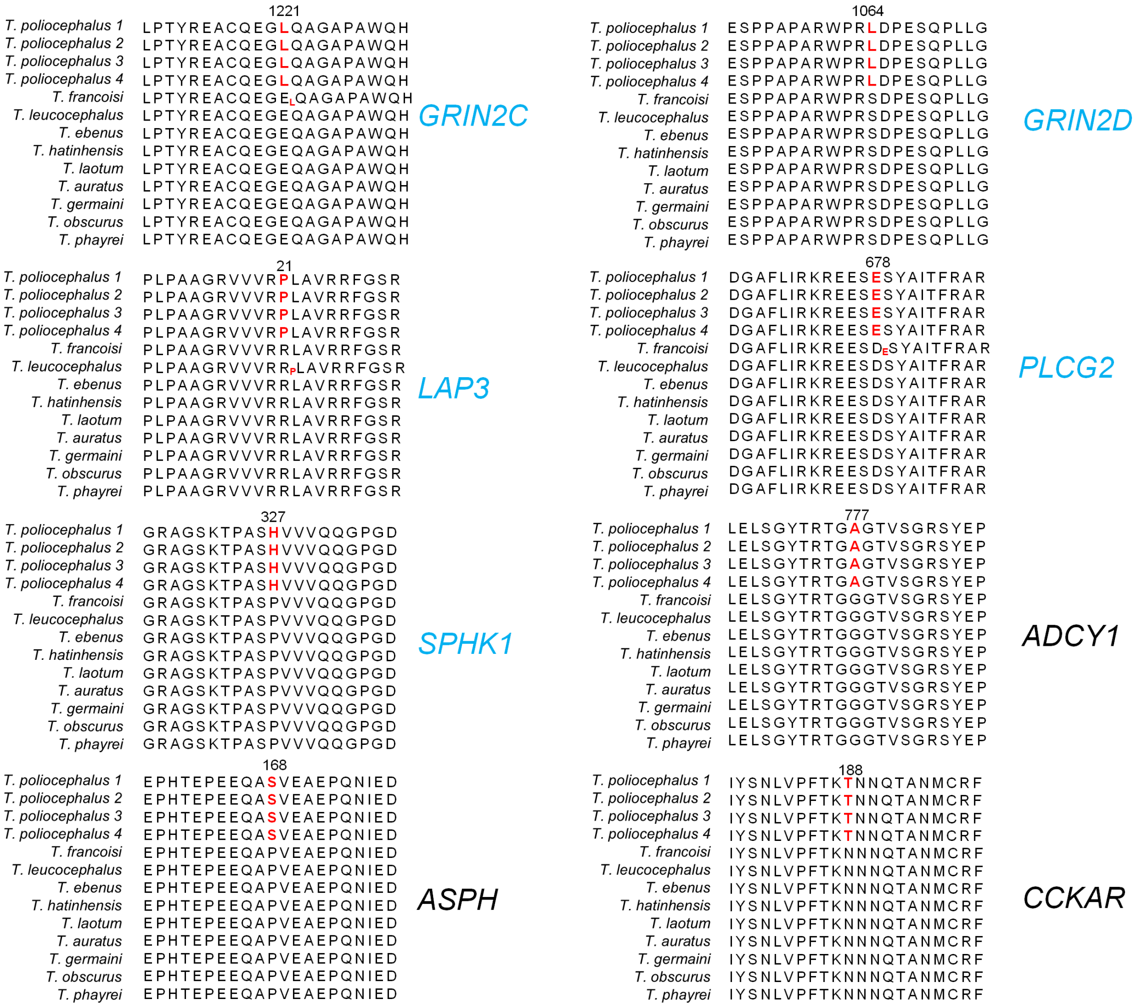

Species	ACKR3 (356)
T. poliocephalus 1	LTKLIDASRVSETEYS**T**LEQSTK
T. poliocephalus 2	LTKLIDASRVSETEYS**T**LEQSTK
T. poliocephalus 3	LTKLIDASRVSETEYS**T**LEQSTK
T. poliocephalus 4	LTKLIDASRVSETEYS**T**LEQSTK
T. francoisi	LTKLIDASRVSETEYSALEQSTK
T. leucocephalus	LTKLIDASRVSETEYSALEQSTK
T. ebenus	LTKLIDASRVSETEYSALEQSTK
T. hatinhensis	LTKLIDASRVSETEYSALEQSTK
T. laotum	LTKLIDASRVSETEYSALEQSTK
T. auratus	LTKLIDASRVSETEYSALEQSTK
T. germaini	LTKLIDASRVSETEYSALEQSTK
T. obscurus	LTKLIDASRVSETEYSALEQSTK
T. phayrei	LTKLIDASRVSETEYSALEQSTK

Species	ACKR4 (290)
T. poliocephalus 1	SKRMDIAIQITE**G**VALFHSCLN
T. poliocephalus 2	SKRMDIAIQITE**G**VALFHSCLN
T. poliocephalus 3	SKRMDIAIQITE**G**VALFHSCLN
T. poliocephalus 4	SKRMDIAIQITE**G**VALFHSCLN
T. francoisi	SKRMDIAIQITESVALFHSCLN
T. leucocephalus	SKRMDIAIQITESVALFHSCLN
T. ebenus	SKRMDIAIQITESVALFHSCLN
T. hatinhensis	SKRMDIAIQITESVALFHSCLN
T. laotum	SKRMDIAIQITESVALFHSCLN
T. auratus	SKRMDIAIQITESVALFHSCLN
T. germaini	SKRMDIAIQITESVALFHSCLN
T. obscurus	SKRMDIAIQITESVALFHSCLN
T. phayrei	SKRMDIAIQITESVALFHSCLN

Species	EDN1 (168)
T. poliocephalus 1	IYQQLVRGRKIR**R**SEEHLRQTR
T. poliocephalus 2	IYQQLVRGRKIR**R**SEEHLRQTR
T. poliocephalus 3	IYQQLVRGRKIR**R**SEEHLRQTR
T. poliocephalus 4	IYQQLVRGRKIR**R**SEEHLRQTR
T. francoisi	IYQQLVRGRKIRSSEEHLRQTR
T. leucocephalus	IYQQLVRGRKIRSSEEHLRQTR
T. ebenus	IYQQLVRGRKIRSSEEHLRQTR
T. hatinhensis	IYQQLVRGRKIRSSEEHLRQTR
T. laotum	IYQQLVRGRKIRSSEEHLRQTR
T. auratus	IYQQLVRGRKIRSSEEHLRQTR
T. germaini	IYQQLVRGRKIRSSEEHLRQTR
T. obscurus	IYQQLVRGRKIRSSEEHLRQTR
T. phayrei	IYQQLVRGRKIRSSEEHLRQTR

Species	EIF2AK3 (770)
T. poliocephalus 1	NLQDSCLTDCD**M**EDGTMDGND
T. poliocephalus 2	NLQDSCLTDCD**M**EDGTMDGND
T. poliocephalus 3	NLQDSCLTDCD**M**EDGTMDGND
T. poliocephalus 4	NLQDSCLTDCD**M**EDGTMDGND
T. francoisi	NLQDSCLTDCDVEDGTMDGND
T. leucocephalus	NLQDSCLTDCDVEDGTMDGND
T. ebenus	NLQDSCLTDCDVEDGTMDGND
T. hatinhensis	NLQDSCLTDCDVEDGTMDGND
T. laotum	NLQDSCLTDCDVEDGTMDGND
T. auratus	NLQDSCLTDCDVEDGTMDGND
T. germaini	NLQDSCLTDCDVEDGTMDGND
T. obscurus	NLQDSCLTDCDVEDGTMDGND
T. phayrei	NLQDSCLTDCDVEDGTMDGND

Species	FPR2 (301)
T. poliocephalus 1	YKIINILVNPTTS**P**AFFNSCLNP
T. poliocephalus 2	YKIINILVNPTTS**P**AFFNSCLNP
T. poliocephalus 3	YKIINILVNPTTS**P**AFFNSCLNP
T. poliocephalus 4	YKIINILVNPTTS**P**AFFNSCLNP
T. francoisi	YKIINILVNPTTSLAFFNSCLNP
T. leucocephalus	YKIINILVNPTTSLAFFNSCLNP
T. ebenus	YKIINILVNPTTSLAFFNSCLNP
T. hatinhensis	YKIINILVNPTTSLAFFNSCLNP
T. laotum	YKIINILVNPTTSLAFFNSCLNP
T. auratus	YKIINILVNPTTSLAFFNSCLNP
T. germaini	YKIINILVNPTTSLAFFNSCLNP
T. obscurus	YKIINILVNPTTSLAFFNSCLNP
T. phayrei	YKIINILVNPTTSLAFFNSCLNP

Species	MCTP2 (866)
T. poliocephalus 1	VQYAELKLCS**R**HSPLRKKRSA
T. poliocephalus 2	VQYAELKLCS**R**HSPLRKKRSA
T. poliocephalus 3	VQYAELKLCS**R**HSPLRKKRSA
T. poliocephalus 4	VQYAELKLCS**R**HSPLRKKRSA
T. francoisi	VQYAELKLCSSHSPLRKKRSA
T. leucocephalus	VQYAELKLCSSHSPLRKKRSA
T. ebenus	VQYAELKLCSSHSPLRKKRSA
T. hatinhensis	VQYAELKLCSSHSPLRKKRSA
T. laotum	VQYAELKLCSSHSPLRKKRSA
T. auratus	VQYAELKLCSSHSPLRKKRSA
T. germaini	VQYAELKLCSSHSPLRKKRSA
T. obscurus	VQYAELKLCSSHSPLRKKRSA
T. phayrei	VQYAELKLCSSHSPLRKKRSA

Species	PLA2G4B (211)
T. poliocephalus 1	QELSIHLQDAP**K**EQLKVPLSA
T. poliocephalus 2	QELSIHLQDAP**K**EQLKVPLSA
T. poliocephalus 3	QELSIHLQDAP**K**EQLKVPLSA
T. poliocephalus 4	QELSIHLQDAP**K**EQLKVPLSA
T. francoisi	QELSIHLQDAPEEQLKVPLSA
T. leucocephalus	QELSIHLQDAPEEQLKVPLSA
T. ebenus	QELSIHLQDAPEEQLKVPLSA
T. hatinhensis	QELSIHLQDAPEEQLKVPLSA
T. laotum	QELSIHLQDAPEEQLKVPLSA
T. auratus	QELSIHLQDAPEEQLKVPLSA
T. germaini	QELSIHLQDAPEEQLKVPLSA
T. obscurus	QELSIHLQDAPEEQLKVPLSA
T. phayrei	QELSIHLQDAPEEQLKVPLSA

Species	LAT2 (146)
T. poliocephalus 1	SYENVLICKQKT**A**EPVAQQDST
T. poliocephalus 2	SYENVLICKQKT**A**EPVAQQDST
T. poliocephalus 3	SYENVLICKQKT**A**EPVAQQDST
T. poliocephalus 4	SYENVLICKQKT**A**EPVAQQDST
T. francoisi	SYENVLICKQKTTEPVAQQDST
T. leucocephalus	SYENVLICKQKTTEPVAQQDST
T. ebenus	SYENVLICKQKTTEPVAQQDST
T. hatinhensis	SYENVLICKQKTTEPVAQQDST
T. laotum	SYENVLICKQKTTEPVAQQDST
T. auratus	SYENVLICKQKTTEPVAQQDST
T. germaini	SYENVLICKQKTTEPVAQQDST
T. obscurus	SYENVLICKQKTTEPVAQQDST
T. phayrei	SYENVLICKQKTTEPVAQQDST

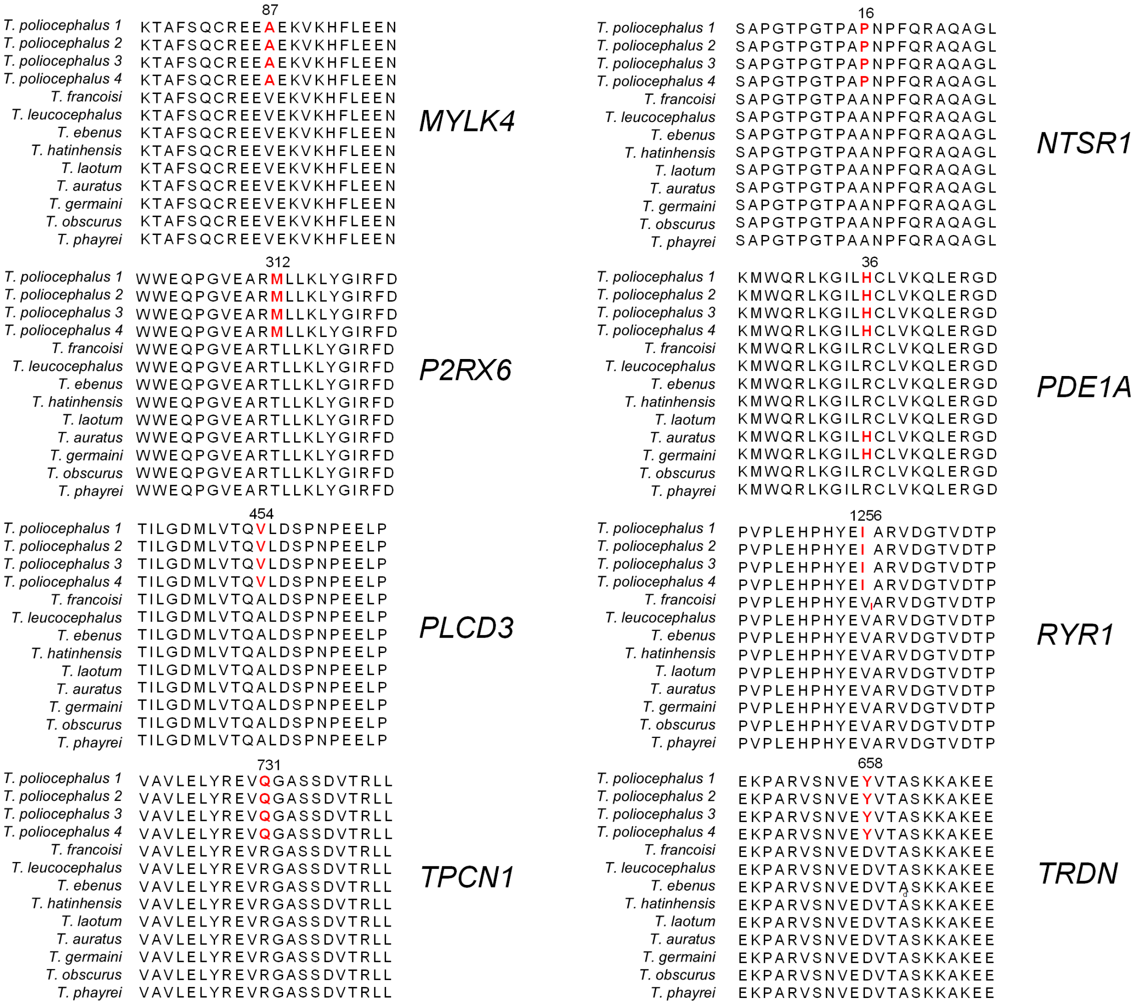

Supplementary Figure 14. Fixed amino acid changes in 24 calcium-related genes in *T. poliocephalus*. Genes in blue represent those found in GO terms “calcium ion binding” and “calcium-mediated signaling”, and KEGG pathway “calcium signaling pathway”. Amino acid changes are shown in red and those in the lower right-hand indicate extremely low frequencies of these amino acids in a heterozygous state in other *Trachypithecus* species.

Supplementary Table 10. Results of functional enrichment analyses related to sodium transportation based on all genes with non-synonymous variants in *T. poliocephalus*.

#Term	Database	ID	Input	Background	*p*-value	Corrected *p*-value
sodium ion transport	GO term	GO:0006814	9	77	0.003138	0.04596
regulation of sodium ion transmembrane transport	GO term	GO:1902305	3	8	0.005702	0.067446
negative regulation of sodium ion transmembrane transporter activity	GO term	GO:2000650	3	9	0.007407	0.079871
sodium ion import across plasma membrane	GO term	GO:0098719	4	19	0.007748	0.081821
ligand-gated sodium channel activity	GO term	GO:0015280	3	10	0.009381	0.092154
sodium ion transmembrane transport	GO term	GO:0035725	9	97	0.012124	0.108478
negative regulation of sodium ion transport	GO term	GO:0010766	2	6	0.029736	0.168973
cellular sodium ion homeostasis	GO term	GO:0006883	3	18	0.035474	0.186395

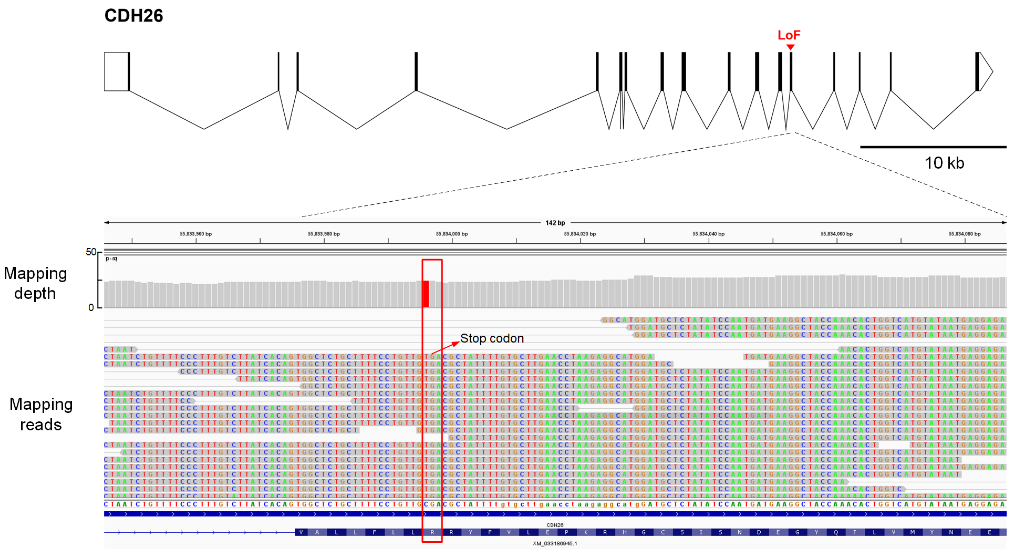

Supplementary Figure 15. Loss-of-function (LoF) variant in *CDH26*.

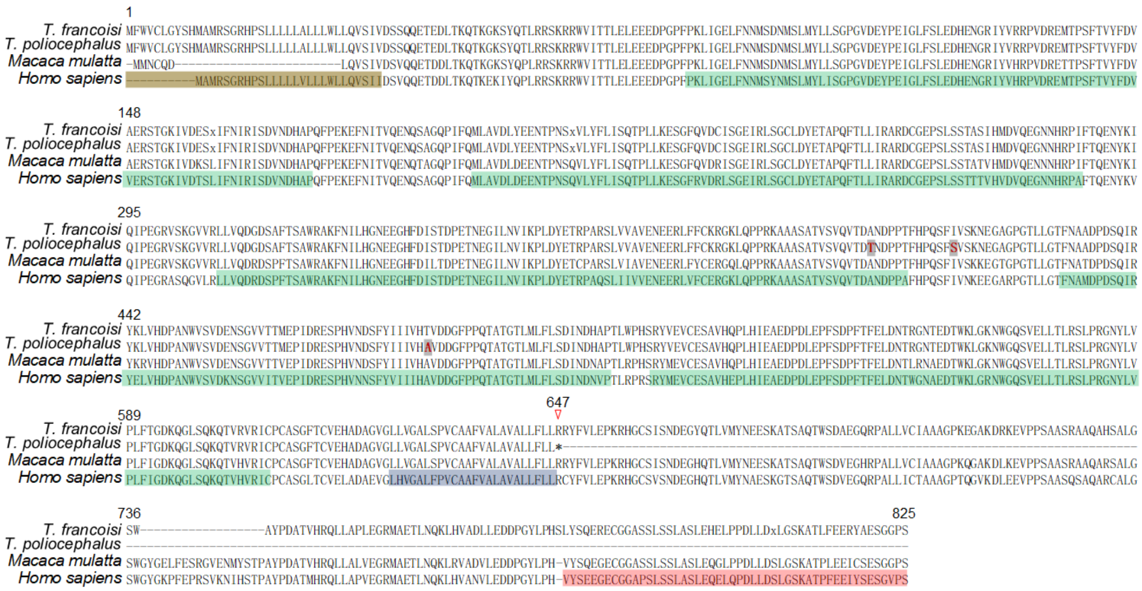

Supplementary Figure 16. Amino acid alignment of CDH26. In *T. poliocephalus* occurs a premature stop codon at position 647. Amino acids in red are unique to *T. poliocephalus*. Brown: signal sequence, green: extracellular cadherin domains (domains are connected via calcium ions), blue: transmembrane region, red: catenin-interacting domain (catenin binds to actin skeleton).

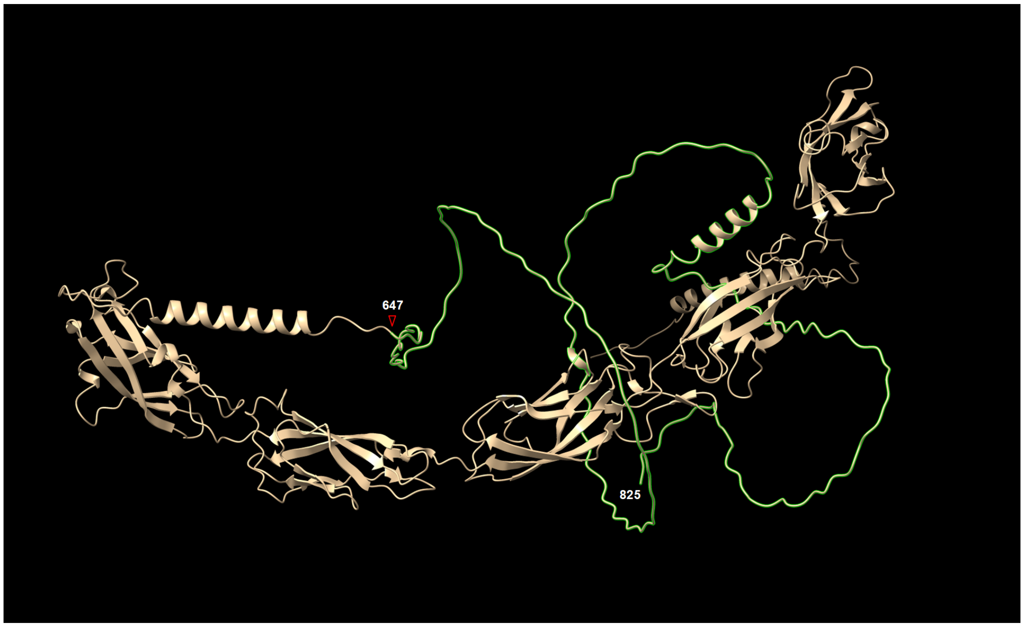

Supplementary Figure 17. Three-dimensional structure of the human CDH26 protein, the cytoplasmatic component, highlighted in green, is lost in *T. poliocephalus*. Image downloaded from UniProt[152] and visualized in ChimeraX[153].

Supplementary Table 11. SNP statistics for the four *T. poliocephalus* samples (Tfra_2.0 reference genome).

Samples	Total SNP	Het SNP	Hom SNP	Non-synonymous	Synonymous
Tpol1	3,860,267	962,512	2,896,175	45,009	44,962
Tpol2	3,854,097	1,004,190	2,848,358	44,634	45,015
Tpol3	3,943,083	1,094,475	2,846,858	45,547	45,046
Tpol4	3,925,636	1,066,267	2,857,751	45,286	44,871

References:

151. Árnason, Ú. et al. Whole-genome sequencing of the blue whale and other rorquals finds signatures for introgressive gene flow. *Sci. Adv.* **4**, eaap9873 (2018).

152. Consortium, UniProt UniProt: the Universal Protein Knowledgebase in 2023. *Nucleic Acids Res.* **51**, D523–D531 (2023).

153. Pettersen, E. F. et al. UCSF ChimeraX: structure visualization for researchers, educators, and developers. *Protein Sci.* **30**, 70–82 (2021).
