# Supplementary material for: Genomic adaptation to small population size and saltwater consumption in the critically endangered Cat Ba langur
Source: Nat Commun. 2024 Oct 2;15:8531. doi: 10.1038/s41467-024-52811-7 (PMC11447269; doi:10.1038/s41467-024-52811-7)
Supplement: Supplementary file 3 — Description of Additional Supplementary Files [file 41467_2024_52811_MOESM3_ESM.pdf]

File Name: Supplementary Movie 1
Description: Cat Ba langurs drinking sea water (video by Nguyen Huy Cam).

File Name: Supplementary Data 1
Description: Heterozygosity ratio in protein-coding versus non-protein-coding regions and ratio of non-synonymous to synonymous variants in protein-coding regions (Mmul_10 reference genome).

File Name: Supplementary Data 2
Description: Number of all alleles and only homozygotes, and ratio comparison of the impact of SNPs on protein-function between *T. francoisi* (Tfra), *T. leucocephalus* (Tleu) and *T. poliocephalus* (Tpol) based on snpEFF annotation (Mmul_10 reference genome).

File Name: Supplementary Data 3
Description: List of homozygous high-impact SNPs (229) and affected genes (220) in all four *T. poliocephalus* individuals (Mmul_10 reference genome).

File Name: Supplementary Data 4
Description: Categories of enrichment analysis with homozygous high-impact genes in *T. poliocephalus*. The corrected *p* values were generated by Benjamini and Hochberg corrected test.

File Name: Supplementary Data 5
Description: List of candidate genes under selective sweeps in *T. poliocephalus* compared to *T. francoisi* (Tfra_2.0 reference genome).

File Name: Supplementary Data 6
Description: Categories of enrichment analysis with selected genes in *T. poliocephalus*. The corrected *p* values were generated by Benjamini and Hochberg corrected test (Tfra_2.0 reference genome).

File Name: Supplementary Data 7
Description: Amino acid changes in all 92 calcium-related genes (including GO terms "calcium ion binding" (71 genes) and "calcium-mediated signaling" (13 genes of which two, *PLA2G4B* and *MCTP2*, overlap with "calcium ion binding") and KEGG pathway "calcium signaling pathway" (16 genes, of which five, *GRIN2C*, *GRIN2D*, *LAP3*, *LAT2* and *SPHK1*, overlap with GO term "calcium-mediated signaling" and one, *RYR1*, overlaps with GO term "calcium ion binding") are fixed in *T. poliocephalus*. Cells highlighted in orange refer to identical homozygous mutations in all four *T. poliocephalus* samples; green and red refers to the same mutation in heterozygous and homozygous state in other *Trachypithecus* individuals, respectively.

File Name: Supplementary Data 8
Description: Amino acid changes in all 22 genes related to sodium transportation (including all GO terms in Supplementary Table 10). Cells highlighted in orange refer to largely fixed homozygous mutations in *T. poliocephalus*, green and red refers to the same mutation in heterozygous and homozygous state in other *Trachypithecus* individuals, respectively.

File Name: Supplementary Data 9

Description: Fixed non-synonymous variants in *T. poliocephalus* compared to other *Trachypithecus* species in salinity-related genes related as reported in previous studies (Tfra_2.0 reference genome).

File Name: Supplementary Data 10

Description: Heterozygosity values (*He*) observed in some mammals.
