# Supplementary material for: Genomic adaptation to small population size and saltwater consumption in the critically endangered Cat Ba langur
Source: Nat Commun. 2024 Oct 2;15:8531. doi: 10.1038/s41467-024-52811-7 (PMC11447269; doi:10.1038/s41467-024-52811-7)
Supplement: Supplementary file 15 — Reporting Summary [file 41467_2024_52811_MOESM15_ESM.pdf]

nature portfolio

Corresponding author(s): Liye Zhang, Michael Hofreiter, Ming Li, Zhijin Liu, Christian Roos

Last updated by author(s): Sep 22, 2024

Reporting Summary

Nature Portfolio wishes to improve the reproducibility of the work that we publish. This form provides structure for consistency and transparency in reporting. For further information on Nature Portfolio policies, see our Editorial Policies and the Editorial Policy Checklist.

Statistics

For all statistical analyses, confirm that the following items are present in the figure legend, table legend, main text, or Methods section.

n/a	Confirmed	
☐	☒	The exact sample size (n) for each experimental group/condition, given as a discrete number and unit of measurement
☐	☒	A statement on whether measurements were taken from distinct samples or whether the same sample was measured repeatedly
☐	☒	The statistical test(s) used AND whether they are one- or two-sided<br>*Only common tests should be described solely by name; describe more complex techniques in the Methods section.*
☒	☐	A description of all covariates tested
☒	☐	A description of any assumptions or corrections, such as tests of normality and adjustment for multiple comparisons
☐	☒	A full description of the statistical parameters including central tendency (e.g. means) or other basic estimates (e.g. regression coefficient) AND variation (e.g. standard deviation) or associated estimates of uncertainty (e.g. confidence intervals)
☐	☒	For null hypothesis testing, the test statistic (e.g. F, t, r) with confidence intervals, effect sizes, degrees of freedom and P value noted<br>*Give P values as exact values whenever suitable.*
☒	☐	For Bayesian analysis, information on the choice of priors and Markov chain Monte Carlo settings
☒	☐	For hierarchical and complex designs, identification of the appropriate level for tests and full reporting of outcomes
☒	☐	Estimates of effect sizes (e.g. Cohen's d, Pearson's r), indicating how they were calculated

Our web collection on statistics for biologists contains articles on many of the points above.

Software and code

Policy information about availability of computer code

Data collection	Detailed information about data collection is presented in the Methods.
Data analysis	Details about data analysis are presented in the Methods.<br>We used the following softwares:<br>sratoolkit v2.9.6; pigz v2.7; fastp v0.23.1; Burrows-Wheeler Aligner v0.7.12; samtools v1.9; Picard v2.20.2; GATK v4.2.2; snpEFF v4.3; King v2.3.2; IQ-tree v2.1.3; FigTree v1.4.0; frappe v1.1136; Admixture v1.3.0; EIGENSOFT v7.2.1; qpDstat; Dtrios & Dinvestigate programs in Dsuite; vcftools v0.1.14; PLINK v1.9; bcftools v1.7; LiftOver; vcfR package; r v4.1.1; tidyverse; KOBAS 3.0; R package rehh; python v2.7; KaKs_calculator v2.0

For manuscripts utilizing custom algorithms or software that are central to the research but not yet described in published literature, software must be made available to editors and reviewers. We strongly encourage code deposition in a community repository (e.g. GitHub). See the Nature Portfolio guidelines for submitting code & software for further information.

Data

Policy information about availability of data

All manuscripts must include a data availability statement. This statement should provide the following information, where applicable:

- Accession codes, unique identifiers, or web links for publicly available datasets
- A description of any restrictions on data availability
- For clinical datasets or third party data, please ensure that the statement adheres to our policy

Newly generated short-read sequencing data are available on NCBI under BioProject PRJNA949813 (https://www.ncbi.nlm.nih.gov/bioproject/949813). Publicly available genome data used in this study can be found under BioProject PRJNA488530 (https://www.ncbi.nlm.nih.gov/bioproject/488530) and in Sequence Read Archive SRR8718596 (https://www.ncbi.nlm.nih.gov/sra/SRR8718596), SRR8718597 (https://www.ncbi.nlm.nih.gov/sra/SRR8718597), SRR1687497 (https://www.ncbi.nlm.nih.gov/sra/SRR1687497), SRR8762000 (https://www.ncbi.nlm.nih.gov/sra/SRR8762000), SRR6196475 (https://www.ncbi.nlm.nih.gov/sra/SRR6196475), SRR16119994 (https://www.ncbi.nlm.nih.gov/sra/SRR16119994), SRR11032814 (https://www.ncbi.nlm.nih.gov/sra/SRR11032814), SRR9703449 (https://www.ncbi.nlm.nih.gov/sra/SRR9703449), SRR11892898 (https://www.ncbi.nlm.nih.gov/sra/SRR11892898), and SRR11075380 (https://www.ncbi.nlm.nih.gov/sra/SRR11075380). Samples of the two deceased Cat Ba langur individuals and the two translocated females are stored at the Cat Ba Langur Conservation Project, Cat Ba Island, Vietnam and the Wildlife Conservation Society, Hanoi, Vietnam, respectively. Source data are provided with this paper.

Research involving human participants, their data, or biological material

Policy information about studies with human participants or human data. See also policy information about sex, gender (identity/presentation), and sexual orientation and race, ethnicity and racism.

Reporting on sex and gender	*Use the terms sex (biological attribute) and gender (shaped by social and cultural circumstances) carefully in order to avoid confusing both terms. Indicate if findings apply to only one sex or gender; describe whether sex and gender were considered in study design; whether sex and/or gender was determined based on self-reporting or assigned and methods used. Provide in the source data disaggregated sex and gender data, where this information has been collected, and if consent has been obtained for sharing of individual-level data; provide overall numbers in this Reporting Summary. Please state if this information has not been collected. Report sex- and gender-based analyses where performed, justify reasons for lack of sex- and gender-based analysis.*
Reporting on race, ethnicity, or other socially relevant groupings	*Please specify the socially constructed or socially relevant categorization variable(s) used in your manuscript and explain why they were used. Please note that such variables should not be used as proxies for other socially constructed/relevant variables (for example, race or ethnicity should not be used as a proxy for socioeconomic status). Provide clear definitions of the relevant terms used, how they were provided (by the participants/respondents, the researchers, or third parties), and the method(s) used to classify people into the different categories (e.g. self-report, census or administrative data, social media data, etc.) Please provide details about how you controlled for confounding variables in your analyses.*
Population characteristics	*Describe the covariate-relevant population characteristics of the human research participants (e.g. age, genotypic information, past and current diagnosis and treatment categories). If you filled out the behavioural & social sciences study design questions and have nothing to add here, write "See above."*
Recruitment	*Describe how participants were recruited. Outline any potential self-selection bias or other biases that may be present and how these are likely to impact results.*
Ethics oversight	*Identify the organization(s) that approved the study protocol.*

Note that full information on the approval of the study protocol must also be provided in the manuscript.

Field-specific reporting

Please select the one below that is the best fit for your research. If you are not sure, read the appropriate sections before making your selection.

☐ Life sciences ☐ Behavioural & social sciences ☒ Ecological, evolutionary & environmental sciences

For a reference copy of the document with all sections, see nature.com/documents/nr-reporting-summary-flat.pdf

Ecological, evolutionary & environmental sciences study design

All studies must disclose on these points even when the disclosure is negative.

Study description	We investigated the evolutionary history, genetic load and adaptive potential of the Cat Ba langur (Trachypithecus poliocephalus), a primate species endemic to Vietnam and with < 100 living individuals one of the most threatened primates in the world.
Research sample	We generated whole-genome sequencing data (range: 28.8–36.5×, mean: 32.7×) from four wild T. poliocephalus individuals representing all three extant sub-populations of the species (Hang Cai, Sanctuary, Cua Dong).

Sampling strategy	No sample size calculation was done. Samples were collected from two translocated females and two deceased infants.
Data collection	Whole blood samples (5 ml) from the two translocated females were collected from the femoral vein, placed in EDTA tubes and kept frozen at –80°C until DNA extraction. Tissue samples were obtained from two deceased infants found in the wild and stored in 80% ethanol until further processing.
Timing and spatial scale	Samples from the two translocated females were taken in 2012 and from the wo deceased infants in 2015 and 2018.
Data exclusions	No data were excluded.
Reproducibility	DNA extraction and whole-genome sequencing methods have been described in the Methods section. The sequencing data, genomic analysis methods, tools used, and their versions have also been provided in the Methods and Data Availability sections for reproducibility.
Randomization	Randomization was not relevant to our study as we conducted a whole-genome sequencing study of a critically endangered primate species.
Blinding	Blinding was not relevant to our study as we conducted a whole-genome sequencing study of a critically endangered primate species.

Did the study involve field work? ☐ Yes ☒ No

Reporting for specific materials, systems and methods

We require information from authors about some types of materials, experimental systems and methods used in many studies. Here, indicate whether each material, system or method listed is relevant to your study. If you are not sure if a list item applies to your research, read the appropriate section before selecting a response.

Materials & experimental systems

n/a	Involved in the study
☒	☐ Antibodies
☒	☐ Eukaryotic cell lines
☒	☐ Palaeontology and archaeology
☐	☒ Animals and other organisms
☒	☐ Clinical data
☒	☐ Dual use research of concern
☒	☐ Plants

Methods

n/a	Involved in the study
☒	☐ ChIP-seq
☒	☐ Flow cytometry
☒	☐ MRI-based neuroimaging

Animals and other research organisms

Policy information about studies involving animals; ARRIVE guidelines recommended for reporting animal research, and Sex and Gender in Research

Laboratory animals	*For laboratory animals, report species, strain and age OR state that the study did not involve laboratory animals.*
Wild animals	Blood samples from two translocated females and tissue samples from two deceased infants provided by the Cat Ba Langur Conservation Project. The blood samples were taken by experienced veterinarians in 2012 during a wild-to-wild translocation aimed at reintroducing two isolated females to the larger of the two breeding sub-populations. The translocation was initially proposed prior to 2008, but the master plan was submitted to relevant Vietnamese authorities and the international community in 2010. The two females were immobilized by delivery of chemical agents (ketamine and medetomidine) via a blow dart after being trapped in a sleeping cave. After initial immobilization they were lowered one by one to the ground by a basket and there intubated and maintained on gaseous anesthesia. Immobilized animals were continuously observed for vital parameters such as respiration, pulse frequency, and internal body temperature. Whole blood samples (5 ml) were collected from the femoral vein, placed in EDTA tubes and kept frozen at –80°C until DNA extraction. Tissue samples from the two deceased infants found in the wild were collected in 2015 and 2018 and stored in 80% ethanol until further processing.
Reporting on sex	*Indicate if findings apply to only one sex; describe whether sex was considered in study design, methods used for assigning sex. Provide data disaggregated for sex where this information has been collected in the source data as appropriate; provide overall numbers in this Reporting Summary. Please state if this information has not been collected. Report sex-based analyses where performed, justify reasons for lack of sex-based analysis.*
Field-collected samples	*For laboratory work with field-collected samples, describe all relevant parameters such as housing, maintenance, temperature, photoperiod and end-of-experiment protocol OR state that the study did not involve samples collected from the field.*
Ethics oversight	Approval to carry out the translocation was granted by HPPC (No: 4398/UBND-NN) and MARD (No: 245/TCLN-BTTN) in early 2010. All research complied with protocols approved by the Animal Welfare Body of the German Primate Center and adhered to the legal requirements of Vietnam. We conducted the study in compliance with the Convention on International Trade in Endangered Species

of Wild Fauna and Flora (CITES, export nr. 18VN0331N/CT-KL, import nr. DE-E-02092/18) and the principles of the American Society of Primatologists for the ethical treatment of nonhuman primates.

Note that full information on the approval of the study protocol must also be provided in the manuscript.

Plants

Seed stocks	*Report on the source of all seed stocks or other plant material used. If applicable, state the seed stock centre and catalogue number. If plant specimens were collected from the field, describe the collection location, date and sampling procedures.*
Novel plant genotypes	*Describe the methods by which all novel plant genotypes were produced. This includes those generated by transgenic approaches, gene editing, chemical/radiation-based mutagenesis and hybridization. For transgenic lines, describe the transformation method, the number of independent lines analyzed and the generation upon which experiments were performed. For gene-edited lines, describe the editor used, the endogenous sequence targeted for editing, the targeting guide RNA sequence (if applicable) and how the editor was applied.*
Authentication	*Describe any authentication procedures for each seed stock used or novel genotype generated. Describe any experiments used to assess the effect of a mutation and, where applicable, how potential secondary effects (e.g. second site T-DNA insertions, mosiacism, off-target gene editing) were examined.*
